# Study on the influences of the fractal dimension of the root system and slope degree on the slope stability

**DOI:** 10.1038/s41598-023-37561-8

**Published:** 2023-06-24

**Authors:** Long Hai, Yongbo Lv, Shilin Tan, Lixin Feng

**Affiliations:** School of Mechanics and Engineering, Liaoning Technical University, Fuxin, 123000 Liaoning China

**Keywords:** Civil engineering, Natural hazards

## Abstract

The problem of quantifying the effect of the alfalfa root morphology on the stability of the shallow surface layer of the slope of the Haizhou open-pit coal mine and the optimal slope degree in terms of the reinforcement of the shallow surface layer by the alfalfa root system was addressed. In this study, the mechanical parameters of plain soil and alfalfa root–soil composite samples were measured by indoor soil tests and triaxial compression tests, and a calculation model for the slope of the Haizhou open-pit coal mine was established in FLAC3D numerical simulation software to analyze the influence of the alfalfa root system on the maximum displacement of the shallow surface layer of the slope and the relationship with the fractal dimension of the alfalfa root system. The fractal dimension was applied to quantify the influence of the alfalfa root morphology to further investigate the relationship between the fractal dimension of the root system and the optimal slope of the shallow surface layer. The analysis showed that the fractal dimension of the alfalfa root system varied at different slope degrees, i.e., 40° > flat > 30° > 50°; the maximum soil displacement of the shallow surface layer of the slope increased with slope in nonlinear increments. Analysis of the fractal dimension of the alfalfa root system and the maximum displacement reduction rate at the different slope degrees revealed that the optimal slope degree of the shallow surface layer reinforced by alfalfa varied between 30° and 40°. The study results could provide a basis for further explaining the nature of the role of the alfalfa root morphology in reinforcing shallow surface soil and the optimal slope degree of the slope of the Haizhou open-pit coal mine reinforced by alfalfa roots.

## Introduction

In recent years, due to the increase in open-pit coal mining, slope reinforcement has received increasing attention. The Haizhou open-pit coal mining site is 4 km long from east to west and 2 km wide from north to south, with a vertical depth of 250 m, which is 18 m lower than sea level, and it went bankrupt in 2005, but the lack of protective measures during coal mining resulted in the formation of a soil‒rock binary structure slope consisting of an upper shallow surface soil layer and a lower weathered bedrock layer; moreover, engineering slope stabilization measures are usually difficult to achieve on soil‒rock dual structure slopes, and improper design or construction can easily lead to geological damage such as landslide slope collapse, which threatens the overall stability of the slope^1–3^; hence, the slope problem cannot be ignored.

Improving the slope stability by optimizing the slope reinforcement process is the goal of many engineering designers. Traditional engineering slope fixing measures can yield a certain slope protection effect at the early stage of the project, but concrete, steel and rocks are constantly eroded under weathering and the effect of rainwater, which leads to a reduction in the protection strength of engineering slope fixing measures. Vegetation plays a role not fulfilled by other engineering measures, such as improving the ecological environment, reducing soil erosion, retaining water and fixing soil and slopes, and vegetation slope fixing is an economic and environmental protection control method with an irreplaceable and important role. Therefore, vegetation slope fixing technology has become the trend and main objective of slope management and maintenance^4^. Manbeian et al.^5^ concluded that the shear strength enhancement effect of root systems on slope soils is influenced by the plant root morphology and plant type and that different plant root morphologies impose different effects on slope stability enhancement. In 1989, Tatsumi^6^ was the first to apply fractal theory to the study of plant root systems, thereby quantifying the morphological and structural characteristics of plant root systems. By studying the fractal characteristics of different plant roots in the desert hinterland, Xiaolin Yang et al.^7^ concluded that the fractal dimension can not only be used to quantify the complexity of root branching but also be considered to predict the biomass of plant roots and thus analyze the soil-fixing capacity of different plants. By analyzing the morphology of the Agriophyllum squarrosum root system under different stand conditions, Jie Ren et al.^8^ concluded that the different stand conditions significantly affected the fractal dimension of the Agriophyllum squarrosum root system. However, this study overlooked the effect of plant roots on the slope stability at different slope degrees. Hongyan Wu et al.^9^ investigated the relationship between the root morphology of Hu Zhi Zi and the stability of slopes at different slope degrees at the Beijing Vulture Peak Forestry station by using the root fractal dimension. However, in this study, only shrub plants were investigated, while the effects of other types of plants on the slope stability were not examined. Currently, numerical simulation is one of the most widely used methods for evaluating the slope stability^10^, and FLAC3D can suitably simulate the three-dimensional mechanical behavior of geotechnical bodies^11^. This algorithm overcomes the assumptions of finite elements and boundary elements based on small deformations, as well as the assumption of discrete elements in treating discrete blocks as rigid bodies^12^, and the model cells can reflect the yield well^13^. Xiaolei Ji^14^ investigated the relationship between the maximum displacement of different plant-reinforced slopes and the fractal dimension of the plant root system through simulation. The plant root morphology has mainly been described by studying the angle between the main and lateral roots of the plant root system, but this method lacks the quantification of the influence of the overall morphology of the plant root system on the stability of the shallow surface layer of the slope. These studies, mostly only considering the soil strength enhancement due to the root system, did not widely considered the effect of the root morphology on the stability of the shallow surface layer of open pit coal mine slopes at different slope degrees and the optimal slope degree of the shallow surface layer of open pit coal mine slopes reinforced by herbaceous plant root systems, but the slope has been employed as a common and important topographic factor^15^; moreover, different slope degrees affect environmental factors such as soil moisture and direct sun angle, which in turn affects the growth of roots and thus the stability of the shallow surface layer of the slope^16^.

Therefore, based on the above studies on the plant root morphology and slope stability, the three aspects of the slope degree, fractal dimension and slope superficial stability were combined and considered to further investigate the optimal slope degree of the surface layer of the slope of the Haizhou open-pit coal mine reinforced by the alfalfa root system by quantifying the relationship between the alfalfa root morphology and the slope superficial stability of the Haizhou open-pit coal mine at different slope degrees.

## Experiment

### Study area

The study site is the northeastern corner of the northern gangue of the open-pit coal mine area in the Haizhou Open-Pit Coal Mine National Mine Park, Fuxin city, Liaoning Province (121°41′1.59"E, 41°59′57.42"N, elevation of 141 m). The Haizhou open-pit coal mine is located in Taiping District, approximately 3 km southeast from the city center of Fuxin city. The Haizhou open-pit coal mine area occurs in the mid-latitude northern temperate zone, with a semihumid and semiarid continental monsoon climate, with average temperatures above 20 °C in summer and below 3 °C in winter, an average annual temperature of 9.5 °C, an average sunshine duration of 2800 h, and a total light energy radiation of 130 kcal/cm^2^^17^. The shallow surface soil of the northeastern corner slope of the northern gangue belongs to the neutral sandy soil developed by the weathering of sandstone in the water spring layer, the lithology is a combination of sandstone, siltstone and coal seams, and the sandstone is interspersed with coal seams of different thicknesses.

### Experimental material

Both the alfalfa root–soil complex and plain soil samples used in the test in this paper comprised in situ soils collected during the same period. The soil quality of the shallow surface layer of the slope was sandy soil, as determined via the indoor sieve analysis method. Compared to remodeled soil, the measured data for in situ soil can better reflect the physical properties of the slope soil because soil remodeling changes the original soil porosity and compaction level, and it is difficult to simulate the difference in soil physical properties between in situ root–soil composites and plain soil in the field^18^, so the measured data for remodeled soil can hardly represent the actual situation and cannot provide data support for the actual project. The calculation parameters of the geotechnical materials of the slope are shown in Table 1.

**Table 1:** Calculation parameters of the geotechnical materials of the slope.

Materials	Density /kg∙m^−3^	Modulus of elasticity /MPa	Poisson’s ratio	Cohesion /kPa	Internal friction angle /°
Plain soil	1700	3	0.3	9.8	24
Alfalfa root–soil complex	1756	3	0.3	12.5	27
Bedrock	2987	6700	0.2	26.8	40

### Experimental method

The cohesive force C and internal friction angle φ of the root–soil composite and plain soil samples were obtained by the indoor direct shear test method; the cohesive force and internal friction angle of the bedrock and the elastic modulus of the three materials were obtained by the triaxial compression test method, and the density of the soil and bedrock was measured by the ring-knife method and sealing method, respectively. Three specimens of each material were prepared for all tests, and the data with a variation of more than 5% were discarded, while the corresponding samples were retested. Then, the three measured data were averaged as the calculation parameters of the geotechnical materials of the slope.

**Figure 1:**
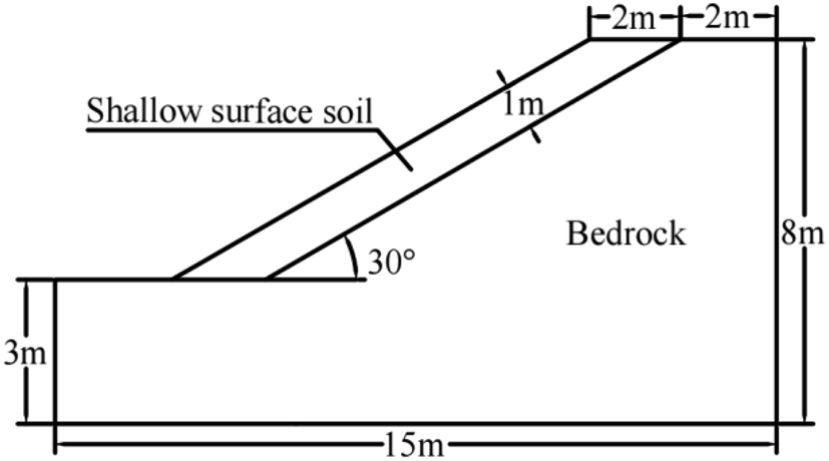
Slope geometry model.

**Figure 2:**
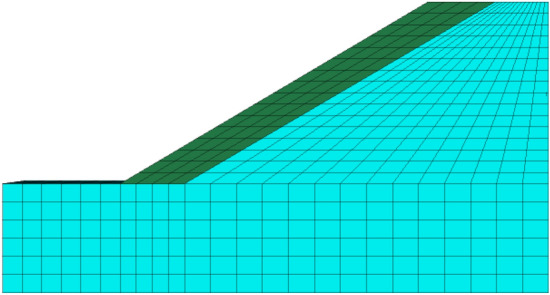
Slope calculation model.

On the side slopes of the study site, the alfalfa root system is a taproot-type system, consisting of primary and lateral roots, and the lateral roots are branch roots arising from the primary root; the roots growing from the lateral roots are called primary lateral roots, the roots growing from the primary lateral roots are called secondary lateral roots, and alfalfa can also produce tertiary lateral roots under the effect of drought stress^19^. Alfalfa generates a well-developed root system, and 1-year-old alfalfa roots can usually reach a depth ranging from 1 to 2 m into the soil^20,21^. The alfalfa root system on the slope of the study site obtained via the excavation method was measured, and the average length of the main roots of 1-year-old alfalfa on each slope was 53.3 cm, with a trend of vertical downward growth. The average length of the lateral roots was 124.6 cm, with a growth trend along all directions starting from 5 cm of the main roots, and the horizontal lateral roots penetrated into the soil to a depth of up to 1 m. Therefore, the calculated model thickness of the shallow surface layer of the slope of the Haizhou open pit was designed as 1 m. The model was created in AutoCAD 2020 software and saved in DXF format according to the slope dimensions, and the specific parameters of the slope geometry model are shown in Fig. 1. The DXF file was imported into FLAC3D 7.0, and the model was stretched by 2 m along the thickness direction to form a preliminary 3D slope model. After the model was established, the mesh was divided, a hexahedral mesh was adopted, and the edge length of a single mesh was set to 0.5 m. The mesh file was generated by using an automatic mesh division program, and the number of mesh cells was 4500 in total, as shown in Fig. 2. Finally, the model was imported into FLAC3D 6.0 for numerical simulation. The model boundary was constrained, with the top surface of the model defined as a free end, the bottom surface was defined as a fixed constraint boundary, and the normal constraint was applied along the perimeter to achieve equilibrium under self-weight.

In this paper, the strength reduction method in the above finite element software was used to calculate the slope safety factor. Since the cohesion C and the internal friction angle φ are the main factors of the slope stability, the coefficient F is was gradually adjusted in the calculation process to obtain different C′ and φ′ values, which are the parameters for repeated iterative calculation. The reduction factor when the slope is in the critical damage state is the safety factor of the slope. The strength reduction method can be expressed as Eqs. (1) and (2).

1 $$C^{^{\prime}} = \frac{1}{F}C,$$

2 $$\phi^{^{\prime}} = \arctan \left( {\frac{\tan \phi }{F}} \right).$$

The cohesion and internal friction angle of the slope in the damage state after discounting can be obtained with Eqs. (1) and (2) by C′ and φ′, respectively. The discounting coefficient is denoted by F, and the cohesion and internal friction angle in the damage state before discounting are denoted by C and φ, respectively. With the use of the built-in FISH language in FLAC 3D software to calculate the slope safety coefficient, in finite element analysis to determine whether the slope reaches the critical instability state, there are three main methods: (1) it is ascertained whether the model calculation process can converge or be fully calculatedcompleted, and when the model calculation process does not converge even when the maximum number of iterations is exceeded, this is determined as slope damage; (2) it is determined whether a large displacement or a displacement inflection point develops over time at a node at the top or foot of the slope as a judgment criterion; (3) it is ascertained whether a continuous plastic through zone emerges from the foot to the top of the slope in the slope calculation model as an evaluation criterion. According to the three convergence judgment criteria mentioned above, the safety coefficient of the slope can be obtained by iterative calculation of the model parameters^22^. In this paper, whether the observed values converged was adopted as a criterion for judging the critical state of slope instability.

### Plant license declaration

The alfalfa plants collected in this experiment were all species commonly encountered around the Haizhou open-pit coal mine in Fuxin city, Liaoning Province, northeastern China, and no valuable plant species were involved, while permission to collect alfalfa plants around the Haizhou open-pit coal mine was obtained. The alfalfa plants were collected in compliance with relevant institutional, national and international guidelines and legislation.

## Results and discussion

### Variation trends of the maximum displacement and safety factor of the shallow surface layer of the slope with slope

**Figure 3:**
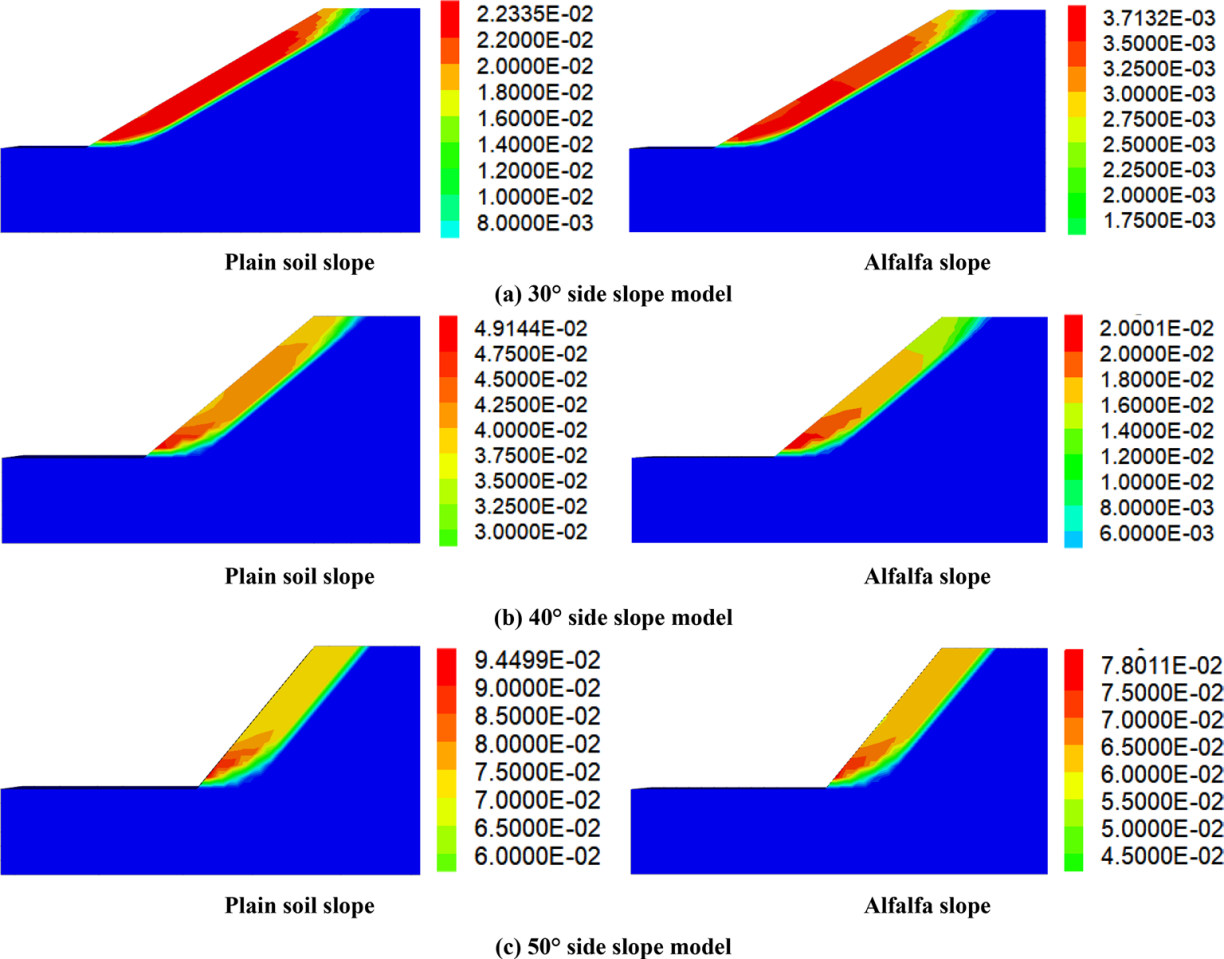
Maximum displacement of the plain soil and alfalfa slopes under the different slope gradients.

In this paper, the effect of alfalfa roots on shallow surface reinforcement of different slopes under the action of self-weight was investigated, and the model considered plain soil slopes and alfalfa slopes with different slopes for simulation calculation. Figure 3 shows that the maximum displacement and displacement cloud range of the alfalfa slopes at the different slope degrees are smaller than those of the plain soil slopes, indicating that the alfalfa root system imposes a positive effect on improving the stability of the shallow surface layer of the slopes. This occurs because the steep slopes exhibit a large water table depth and a low vegetation cover and biodiversity, the weathering effect mainly involves physical weathering, and the weathering effect of surface soil is weak. However, the weathering products are easily washed away and eroded. In contrast, on the gentle slopes, due to the flatter terrain, frequent surface water and groundwater processes, higher vegetation cover and biodiversity, and predominance of chemical weathering, the weathering effect of the surface soil is stronger, and the weathering products are less eroded by scouring than those on the steep slopes. Therefore, the range of the maximum displacement occurring on the gentle slopes is larger than that on the steep slopes, but the maximum displacement on the steep slopes is larger than that on the gentle slopes because the sliding force on former is greater than that on the latter and the stress is concentrated at the foot of the slope^23,24^. This indicates that the steeper the slope is, the more pronounced the role of the alfalfa root–soil complex in reinforcement of the shallow surface layer^25^.

According to the analysis of the data in Table 2, the safety coefficient of the alfalfa slopes is higher than that of the plain soil slopes at the various slope degrees, and the increase rate of the safety coefficient at slopes of 30°, 40°, and 50° is approximately 17%. This occurs because the mechanical properties of roots are tensile but not compressive, whereas soil is compressive but not tensile. Combining the two materials to form root–soil composites can supplement the deficiencies of both and effectively improve the stability of the shallow surface layer of the slope. The maximum displacements of the alfalfa slopes were smaller than those of the plain soil slopes at slopes of 30°, 40° and 50°. Compared to those of the plain soil slopes, the maximum displacements of the 30° slopes were reduced by 83.0%, the maximum displacements of the 40° slopes were reduced by 59.3%, and the maximum displacements of the 50° slopes were reduced by 17.4%.

**Table 2:** Simulation results for the different types of slopes.

Slope/°	Maximum displacement of the plain soil slopes/mm	Factor of safety of the plain soil slopes	Maximum displacement of the alfalfa slopes/mm	Alfalfa slope safety factor
30	22.34	2.10	3.71	2.46
40	49.14	1.52	20.00	1.77
50	94.50	1.20	78.01	1.40

**Figure 4:**
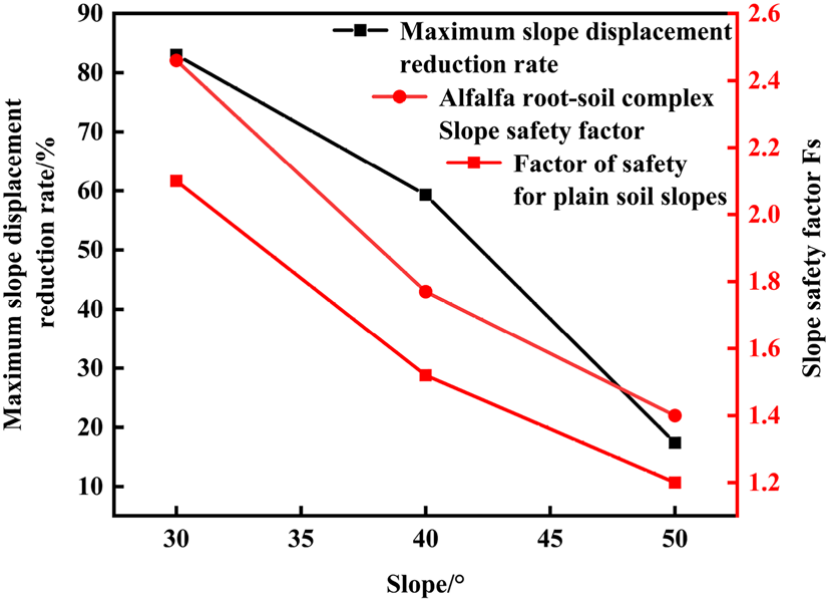
Relationship between the slope maximum displacement reduction rate and slope safety factor.

Figure 4 shows the relationship between the maximum displacement reduction rate and the slope safety coefficient of the slopes at the different slope degrees, and the maximum displacement reduction rate gradually decreases with increasing slope because when the steep slope is subject to drought stress exceeding the tolerance range of the alfalfa root system, the growth of the primary root is inhibited, and the lateral roots mainly provide soil reinforcement. However, the growth of the lateral roots is limited by their inherent strength, so the role of alfalfa in increasing the slope stability gradually decreases with increasing slope degree. The growth rate of the slope safety factor did not change significantly. This occurs because the safety coefficient of each slope is above 1, which suggests that the slope is in a relatively safe state and the reduction in the maximum displacement of the slope by the root system is limited. As the root system of alfalfa provides natural reinforcement, root–soil composites can be regarded as reinforced soil. The distribution of the alfalfa root system in soil is more complicated than that of traditional engineering reinforcing materials, with a large diameter of the main root, which plays the role of anchoring in soil, and a small diameter and large number of lateral roots, which form net-like pockets in soil and mainly provide reinforcement. Alfalfa roots implanted in the superficial layer of the slope can share part of the stress of the soil body and transfer the stress to the surrounding soil body through the primary and lateral roots, thus increasing the ability of the root system implanted in the superficial layer of the soil body to resist external loads, improving the shear strength of the superficial layer of the slope, enhancing the stability of the slope and significantly increasing the safety factor^14^.

### Relationship between the fractal dimension of the root system and maximum displacement of the slope

**Figure 5:**
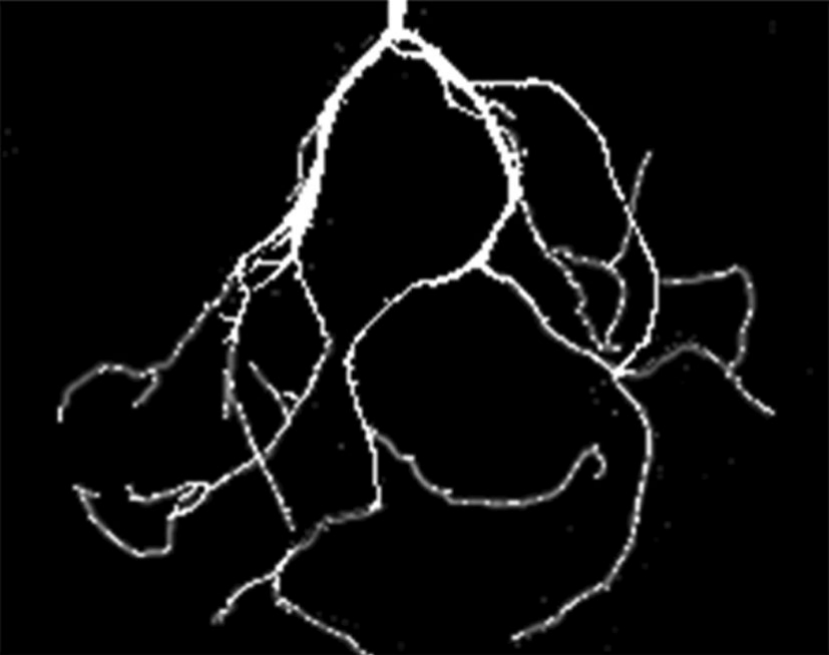
Binary image of the alfalfa root morphology.

Three one-year-old alfalfa plants were selected on each slope, and the roots were gently separated from the soil using a brush. Regarding the treated root system, the root system was scanned along four directions using an HP P8700 scanner, and the root morphology was extracted from the scanning results using WinRHIZO Tron software^26^. The extracted alfalfa root morphology images were processed in FracLab 2.2 software to obtain binary images, as shown in Fig. 5. The root fractal dimension of the binary images was calculated, and the average value was obtained. The average fractal dimension of the root system of the three alfalfa plants selected from the same batch on the same slope was calculated as the average fractal dimension of the root system on the considered slope, and the average fractal dimension of the root system at the different slope degrees was calculated separately by this method.

**Figure 6:**
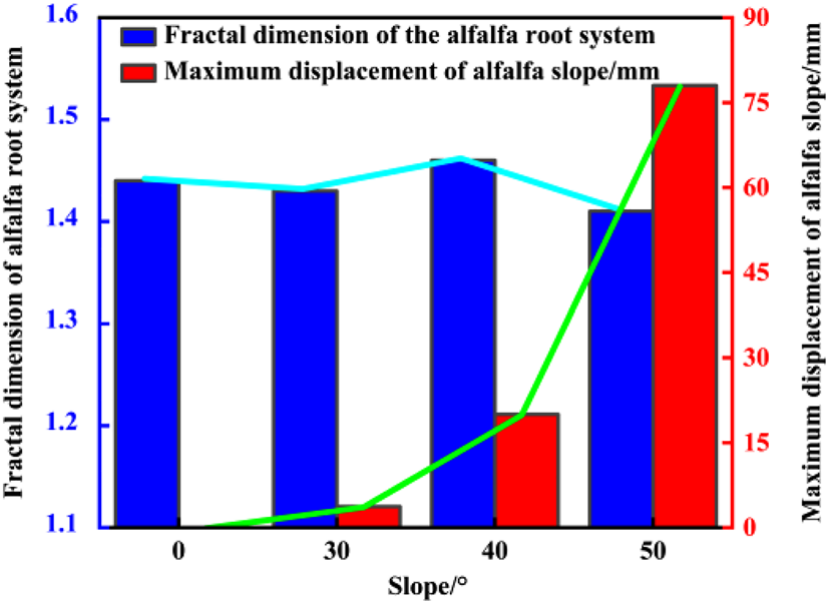
Fractal dimension of the alfalfa root system and maximum displacement of slope at the different slope degrees.

According to Table 3, the root fractal dimension was the highest on the 40° slopes, rising by 1.4%, 2.1%, and 3.5%, respectively, relative to the slopes of 30° and 50°, while it was marginally lower on the 30° slopes than flat land. The fractal dimension of the root system steeply decreases when the slope exceeds 40°, which is consistent with the finding of Hao Wang et al.^27^ indicating that the overall fractal dimension of the sea buckthorn root system first increases and then decreases at different slope degrees. The relationship between the fractal dimension of the alfalfa root system and the maximum displacement of the shallow surface layer of the slope under the different slope degrees is shown in Fig. 6. It is accepted that the growth of plant roots requires water retention on the slope, and the water content in the slope is one of the main factors affecting the morphology of plant roots^28^. Since the 30° side slope provides a better hydrophobic effect than flat land, the soil nutrient loss from the side slope occurs slightly faster, resulting in lower drought stress on the plant roots. The difference in the fractal dimension of the alfalfa root system between the two site conditions is small, so the maximum displacement of the 30° side slope is not obvious. With increasing slope, the soil erosion of the slope increases, the plant root system is subject to higher drought stress, and the water retained in the slope cannot fully satisfy the normal plant growth requirements, so the root system grows many lateral roots horizontally and vertically to absorb nutrients along all directions and simultaneously increases the contact area between the root system and soil particles, improves the shear strength of the soil body in the shallow surface layer of the slope, and reduces the maximum displacement of the shallow surface layer of the slope, thus increasing the stability of the soil body in the superficial surface layer of the slope, which is consistent with the findings of Wenyao Li et al.^29^ indicating that drought stress promotes the growth of lateral alfalfa roots, which in turn improves the stability of the slope. When the slope gradient exceeds 40°, the resulting slope erosion is serious, the slope drought stress increases, and the tangential stress on the plant root system on high and steep slopes is greater than the strength of the root system itself. Therefore, the fine lateral roots are mainly broken and cannot grow, and only the primary root can provide a reinforcing role, resulting in a reduction in the fractal dimension of the alfalfa root system. The decrease in soil cohesion of the shallow surface layer of the slope results in a reduction in the shear strength of the shallow surface layer, and the maximum displacement of the slope substantially increases, lowering the stability of the shallow surface layer of the slope^30^. The underlying mechanism may be related to changes in the root plasticity due to drought stress and continuous adaptation to the environment^31^.

**Table 3:** Fractal dimension of the alfalfa root system under the different site conditions.

Alfalfa standing conditions	Average fractal dimension of the root system
Flat land	1.44
30° side slopes	1.43
40° side slopes	1.46
50° side slopes	1.41

### Study of the optimal slope degree of shallow surface reinforcement by the alfalfa root system

**Figure 7:**
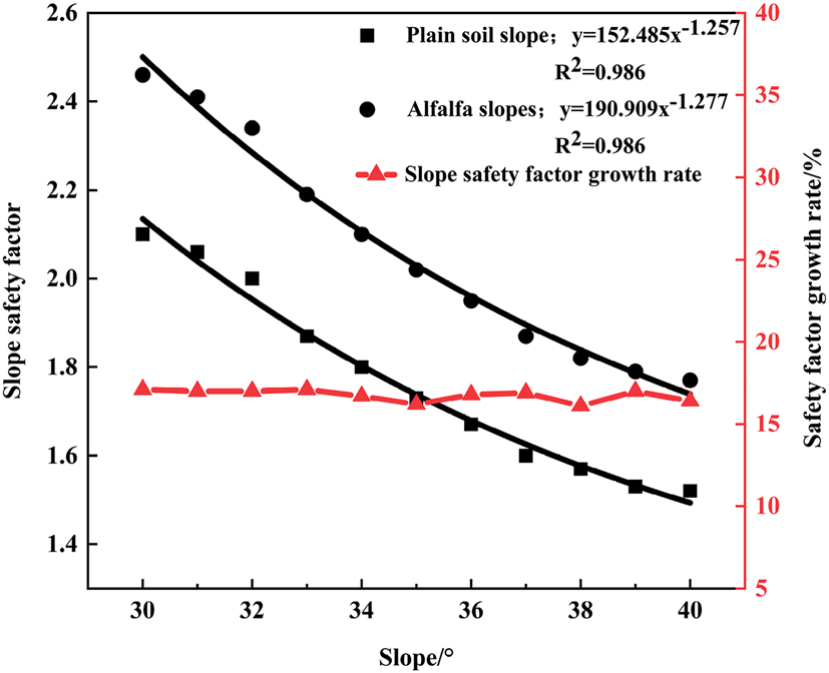
Variation trend of the safety coefficient of the 30° ~ 40° slopes.

**Figure 8:**
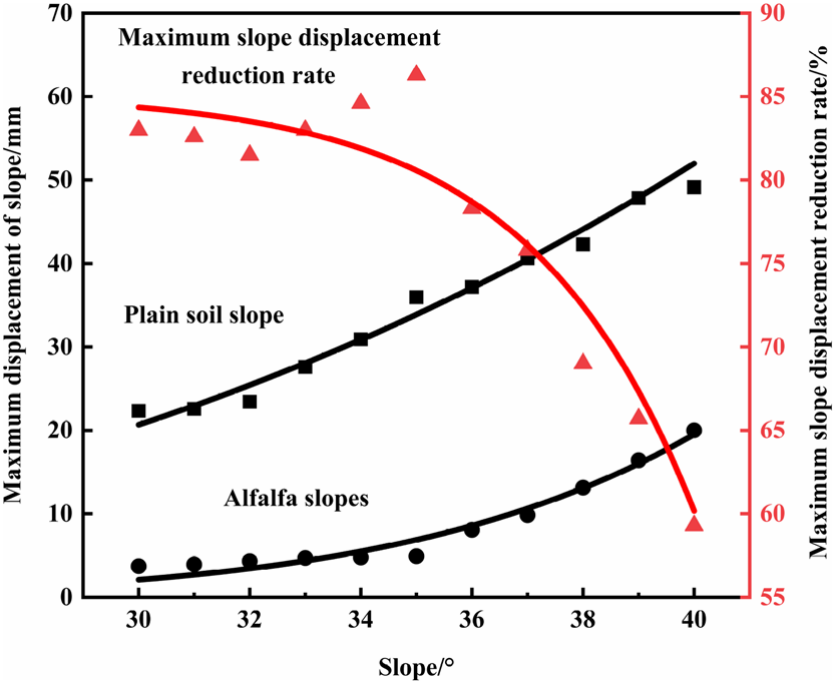
Variation trend of the maximum displacement of the 30° ~ 40° slopes.

The analysis of Table 2 reveals that the maximum displacement reduction rate is 23.7% higher for the 30° slopes than for the 40° slopes and 41.9% higher for the 40° slopes than for the 50° slopes, and the fractal dimension of the alfalfa root system is the highest for the 40° side slope. The results show that the optimal slope degree range is 30° ~ 40° for the alfalfa root system to reinforce the shallow surface layer of the slope. FLAC3D software is employed to simulate various slope models between 30° and 40° with increments of 1°, and the results are provided in Table 4 as the maximum displacement, safety factor, and growth rate of the safety factor of the vegetated soil slopes and alfalfa slopes under each slope gradient. Figure 7 shows the variation trend of the safety coefficients of the plain soil and alfalfa slopes under a 30° ~ 40° slope gradient. It can be found that the slope and slope safety coefficient exhibit a power function relationship and that the slope safety coefficient declines with increasing slope. The slope and safety factor fitting equations are as follows: plain soil slope: y = 152.485x^−1.257^, R^2^ = 0.986; alfalfa slope: y = 190.909x^−1.277^, R^2^ = 0.986. Compared to that of the plain soil slope, the growth rate of the safety factor of the alfalfa slope is not significantly different from that of the slope studied above. This is due to the limitation of the growth rate of the safety factor of the 30° and 40° slopes and the small slope increment of the 30° to 40° slope calculation models, so the growth rate of the safety factor of the slope is approximately 17%. Figure 8 shows the variation pattern of the maximum displacement of the alfalfa slopes between 30° and 40°. The maximum displacement increases with increasing slope as a power function. The fitting equation of the maximum displacement and slope is as follows: y = 3.799·10^−4^x^3.206^, R^2^ = 0.986; alfalfa slope: y = 6.571·10^−12^x^7.785^, R^2^ = 0.958. The reduction rate of the maximum displacement decreases exponentially with slope, and the fitting equation is y = 85.212–3.514·10^−5^e^0.337x^, R^2^ = 0.902. When the slope is 35°, the maximum displacement reduction rate reaches its maximum value of 86.3%, indicating that this slope is the optimal slope degree. The maximum displacement of both the plain soil and alfalfa slopes gradually increases with increasing slope, and the maximum displacement reduction rate gradually decreases.

**Table 4:** Stability parameters of the 30° ~ 40° plain soil and alfalfa slopes.

Slope/°	Factor of safety of the plain soil slopes	Maximum displacement of the plain soil slopes/mm	Alfalfa slope safety factor	Maximum displacement of the alfalfa slopes/mm	Safety factor growth rate /%
30	2.10	22.34	2.46	3.71	17.1%
31	2.06	22.57	2.41	3.92	17.0%
32	2.00	23.43	2.34	4.33	17.0%
33	1.87	27.59	2.19	4.70	17.1%
34	1.80	30.92	2.10	4.76	16.7%
35	1.73	35.95	2.02	4.92	16.2%
36	1.67	37.19	1.95	8.08	16.8%
37	1.60	40.63	1.87	9.83	16.9%
38	1.57	42.29	1.82	13.13	16.1%
39	1.53	47.85	1.79	16.42	17.0%
40	1.52	49.14	1.77	20.00	16.4%

## Discussion

### Fractal dimension of the alfalfa root system and slope stability

The existing studies on vegetation slope stabilization mainly focus on numerical analysis of the interaction mechanism between plant roots and soil, the mechanical properties of plant roots and the single factors of the plant root morphology, such as the number, diameter, length and angle of the main and lateral plant roots. The plant root morphology imposes a significant effect on the reinforcement of the shallow surface layers of slopes, but there are few studies on the application of the fractal dimension to quantify the plant root morphology and its effect on the stability of shallow surface layers at different slope degrees. In this paper, a numerical simulation model for the slope of the Haizhou open-pit coal mine was established by analyzing field research and available indoor test data. Compared to existing studies, WinRHIZO Tron software is used in this study to obtain the complete alfalfa root system, and FracLab2.2 software is employed to obtain the fractal dimension of the whole alfalfa root system, which is combined with the maximum displacement and safety factor of the slope to further investigate the influence of the fractal dimension of the alfalfa root system on the stability of the shallow surface layer of the slope.

### Optimal slope degree for shallow surface reinforcement of slopes by the alfalfa root system

In this study, one-year-old specimens of alfalfa were used to investigate the effect of the alfalfa root morphology on the slope stabilization effect of the shallow surface layer of the Haizhou open-pit coal mine. Via analysis of the fractal dimension of the alfalfa root system, it was found that there existed an optimal slope degree for the alfalfa root system in regard to reinforcing the shallow surface layer of the slope of the Haizhou open-pit coal mine due to the strength of the alfalfa root system itself. The optimal slope degree range was determined from the fractal dimension of the alfalfa root system, the maximum displacement of the shallow surface layer of the slope and the safety coefficient of the slope, and a numerical simulation model for the slope was established under different slope degrees (in increments of 1 degree) to determine the optimal slope degree for reinforcing the shallow surface layer of the slope of the Haizhou open-pit coal mine by the alfalfa root system, which reflects the important ecological value of alfalfa for slope management of the Haizhou open-pit coal mine.

## Conclusions

In this paper, by establishing a finite element model for calculating soil‒rock binary structure slopes of the Haizhou open-pit coal mine under different slope degrees, the maximum displacement of the shallow surface layer of plain and alfalfa slopes, the safety factor and the fractal dimension of the plant root system can intuitively and scientifically reflect the change pattern of the soil reinforcement effect of the plant root system with slope, and the following conclusions are obtained after analysis and discussion:

1. The maximum displacements of the plain soil and alfalfa slopes are consistent with the change in slope, both increasing as a power function. The rate of reduction of the maximum displacement of the topsoil layer on the alfalfa slopes relative to the plain soil slopes decreases as an exponential function (y = 85.212–3.514·10^−5^e^0.337x^, R^2^ = 0.902), and the higher the slope gradient, the smaller the effect of the alfalfa root–soil complex on slope stability improvement is. The slope safety coefficient exhibits a power function decreasing relationship with the slope gradient. The growth rate of the safety coefficient of the alfalfa slope relative to the plain soil slope is approximately 17% in both cases.
2. The fractal dimension of the alfalfa root system first increases and then decreases with slope at the different slope degrees, among which the fractal dimension of the root system is the highest at 40°, indicating that when alfalfa is subject to drought stress, it is more favorable for the lateral roots to produce net-like pockets and improve the stability of the slope. When the slope is greater than 40°, the number of lateral roots of the alfalfa root system decreases due to its strength and severe drought stress, and the fractal dimension of the alfalfa root system decreases.
3. The optimal slope degree for the shallow surface layer of the slope reinforced by the alfalfa root system varied between 30° and 40°. The study relied on numerical simulations, and it was discovered that the maximum displacement of the alfalfa slope was 86.3% smaller than that of the plain soil slope at the 35° slope gradient. The results showed that a 35° slope of the Haizhou open-pit coal mine is the optimal slope degree for reinforcing the shallow surface layer of the slope by alfalfa.

Finally, to study the influence of the alfalfa root morphology on the stability of the shallow surface layer of the slope of the Haizhou open-pit coal mine, the influence of regional rainfall on the displacement of the shallow surface layer and the overall safety factor of the slope must be carefully considered.

## Data Availability

The datasets used and/or analyzed in this study are available from the corresponding author upon reasonable request.
